# The efficacy of a mobile phone application to improve adherence to treatment and self-management in people with chronic respiratory disease in Romanian population – a pilot study

**DOI:** 10.1186/s12913-020-05340-0

**Published:** 2020-05-27

**Authors:** Laura Adela Munteanu, Mirela Frandes, Bogdan Timar, Emanuela Tudorache, Ariadna Petronela Fildan, Cristian Oancea, Doina Ecaterina Tofolean

**Affiliations:** 1 Department of Pulmonology, “Victor Babeș” University of Medicine and Pharmacy, Timișoara, Romania; 2 Department of Biostatistics and Medical Informatics, “Victor Babeș” University of Medicine and Pharmacy, Eftimie Murgu, 30041 Timișoara, Romania; 3 Internal Medicine Discipline, Medical Clinical Disciplines I, “Ovidius” University of Constanta, Faculty of Medicine, Constanta, Romania

**Keywords:** Asthma, M-health, Quality of life, Chronic disease, Exacerbation

## Abstract

**Background:**

Many studies assessed the effect of mobile phone applications on self-management outcomes in patients with asthma, but all of them presented variable results. In this paper. we examined the effect of a mobile phone application on self-management and disease control in Romanian population.

**Methods:**

This study included 93 patients diagnosed with asthma that were recalled every three months for a year for assessment and treatment. Patients were divided into two groups. The first group included patients that received treatment, and the second group received treatment and also used the smartphone application. Number of exacerbations and asthma control test (ACT) were recorded.

**Results:**

The ACT score was significantly higher for asthma patients using also the mobile application than for the patients using the treatment alone, for all the evaluation moments (Mann-Whitney U test, *p* <  0.001). Also, we found significant differences between the ACT score with-in each group, observing a significant improvement of the score between evaluations and baseline (related-samples Friedman’s test with Bonferroni correction, *p* <  0.001). When considering the exacerbations rate, significantly less patients using the application presented exacerbations, 10.30% vs. 46.30% (Pearson Chi-square test, X^2^ (1) = 13.707, *p* <  0.001).

**Conclusion:**

Our study indicates that smartphone applications are an effective way to improve asthma control and self-management when used continually in our population. We found significant positive effects in disease control and exacerbation frequency.

## Background

Asthma is a chronic respiratory disease that affects more than 334 million people of all ages in all parts of the world. It is estimated that the number of patients suffering of asthma will increase by 100 million more until 2025 [1].

Although there are many evidence-based guidelines and effective treatments for this disease, many patients with asthma still have uncontrolled symptoms. Guidelines define poor control of the disease as having daytime symptoms, the need to use short-acting inhaler more than twice weekly, having reduced activities due to asthma or having to wake up due to the respiratory symptoms [1].

There is significant evidence for the effectiveness of self-management in asthma but there are important challenges implementing this support. These obstacles appear due to the patients lack of knowledge and training regarding the disease and inhaler technique and due to the healthcare provider’s lack of time, skill or motivation to support self-management in patients [2, 3].

Over the last three decades important improvements have been made regarding the adherence to treatment in patients with asthma with the help of the Internet and other electronic modalities [4, 5].

In the late 90’s the concept of electronic management of health (e-health) appeared, defining an area at the intersection of public health, medical informatics and business with the objective to improve and deliver medical information and services [6].

There are two modalities by which healthcare can be provided with the help of technology. The first one is Tele-health which means providing healthcare “at a distance” and “digital interventions” which means delivering healthcare by web-based interventions on PC or mobile devices, applications (app) on smartphone, etc. [7].

A recent research found that the key components to improve asthma outcome with the help of mobile devices are: information and self-care education, self-monitoring, asthma action plans, feedback from devices, alerts and messages to patients and daily use availability [8].

Estimates show that by 2019 more than a billion people will use m-Health apps on their smartphones [9].

Due to the fact that these m-Health app are an easy way to communicate information, share experiences and are low cost and easily available, they can have the potential to improve self-management in patients with asthma [10, 11].

Many studies assessed the effects of m-Health app on self-management outcomes in patients with asthma, but all of them presented variable results [12–15].

Some of them demonstrated that m-Health app can improve pulmonary function, asthma symptoms, quality of life, reduce the rate of exacerbations [16], and others found no significant impact on the above mentioned parameters [15].

Although a meta-analysis questioned the evidence behind the effectiveness of telemedicine interventions in asthma [17], a recent systemic review conducted on digital interventions for asthma presented promising conclusions [8].

In a study that evaluated the predictors of uncontrolled bronchial asthma, 58% of the studied patients were found with exacerbations due to different reasons [18].

The European Academy of Allergy and Clinical Immunology (EAACI) analysed 136 mobile phone app to depict a broad heterogeneity in terms of quality and content. Regarding the app for asthma, Matricardi et al. observed that these had the potential to enhance quality of care, improve adherence to treatment and detect modifications of symptoms through continuous monitoring and feedback to patients [19].

Moreover, in a recent review of mobile phone app that support self-management for people with asthma, authors concluded that app that incorporate an action plan and other self-monitoring features, can be an effective option for supporting self-management [20].

The conclusions of the last Romanian awareness campaign on the World Asthma Day showed that treating asthma is deficient, just 13.5% of the studied patients known as diagnosed with asthma being actually treated [21].

Although nowadays there are many mobile phone app designed to improve healthcare, there are no specially designed app for asthma patients in Romania. In Romania asthma remains underdiagnosed and, in many cases, poorly treated thus the objective of this paper was to observe the effect of a mobile phone app on self-management and disease control in Romanian population.

## Methods

### Design

All the subjects have been informed upon the research, and written informed consent was obtained before the beginning of the study. This research was respecting the Declaration of Helsinki ethical principles for research regarding the safety of human subjects. The study design and contract forms were approved by the Ethics Committee of the “Victor Babes” hospital (nr.8068/20/09/2018).

We included patients meeting the criteria of the Global Initiative for Asthma (GINA) guidelines (history of characteristic symptom patterns and variable airflow limitation FEV_1_/FVC < 0.7 and FEV_1_ increases by > 200 mL and > 12% of the baseline value) [1] who were unable to control the condition although they received proper treatment and age > 18 years (this was considered the first evaluation = T0). The patients were selected from the general population who came to routine control in our clinic.

Exclusion criteria were: illiteracy, cognitive impairment, no knowledge how to properly use a smartphone, other pulmonary obstructive diseases, asthma-COPD overlap syndrome (ACO).

This was a pragmatic non-randomized controlled trial. The selected patients were from the general population. They were recruited when they visited the clinic for asthma symptoms. For a period of 6 months we included all the patients that were diagnosed with asthma and were willing to participate in the study. According to our study design, at the T0 visit (which we considered the baseline value), patients were evaluated and trained regarding the inhalation technique. All the patients received at this point an action plan that was explained face to face and have been instructed regarding the inhaler technique, afterwards, the patients were divided into two groups. The separation was made according to the will of the patient to use the app and availability or not of a smartphone. The first group included 54 patients that received only pharmacological treatment and the second group that consisted out of 39 patients received treatment and used the smartphone application. The mobile phone app reminded daily the patients to use the prescribed medication and to complete the specific questionnaire. A graph was daily created by the program once the patients introduced the values and the physician could observe the results in real time. The patients within the control group were allocated by matching them with those from the group using the mobile application.

We excluded from the study the patients that did not access the application when reminded and did not follow the instructions (in total 12 patients were excluded). Excluded patients stated as motivation of not using the application as time consuming or hard to use.

### Measures and instruments

The included patients were afterwards recalled every 3 months for 1 year for assessment and treatment (T1 to T4). Number of exacerbations and asthma control test were recorded. Patients completed every recall the asthma control test questionnaire (ACT) which is used to assess the symptoms. This questionnaire is a patient self-administered tool for identifying those with poorly controlled asthma. Its score ranges from 5 to 25 (the higher score the better). Scores ranging 20–25 are classified as well-controlled, scores from 16 to 19 represent not well controlled and 5–15 as very poorly controlled. The minimally important difference is 3 points between two groups. The medication step-up and step-down was made according to the GINA guideline [1].

Exacerbations are defined as acute episodes, which are characterized by progressive increase in one or more typical asthma symptoms (dyspnea, cough, wheezing and chest tightness) accompanied by a decrease in expiratory flow [1].

**Fig. 1:**
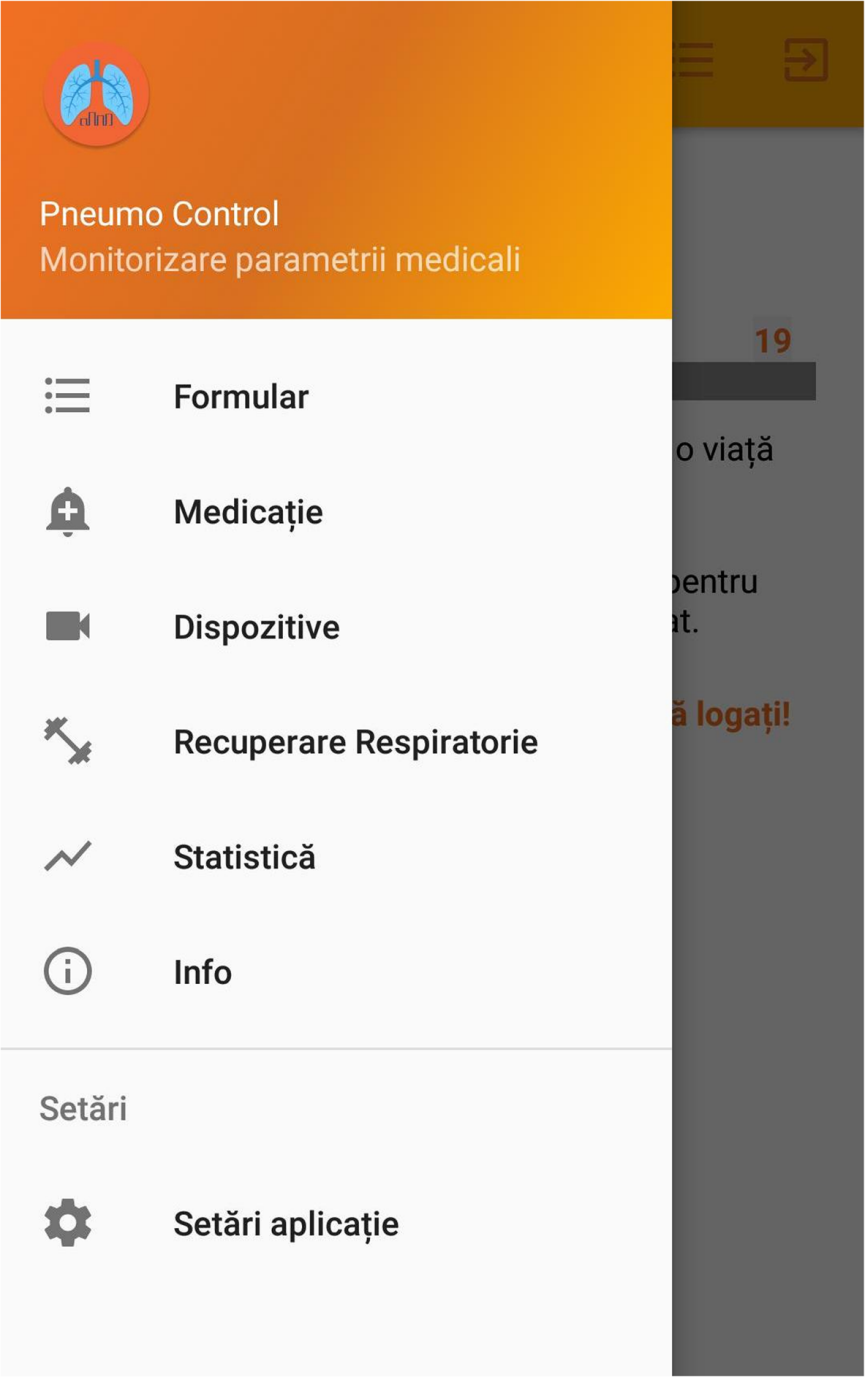
Application menu. Formular – form. Medicatie – medication. Dispozitive – devices. Recuperare respiratory – pulmonary rehabilitation. Statistica – statistics. Info – information Setari aplicatie – application settings

**Fig. 2:**
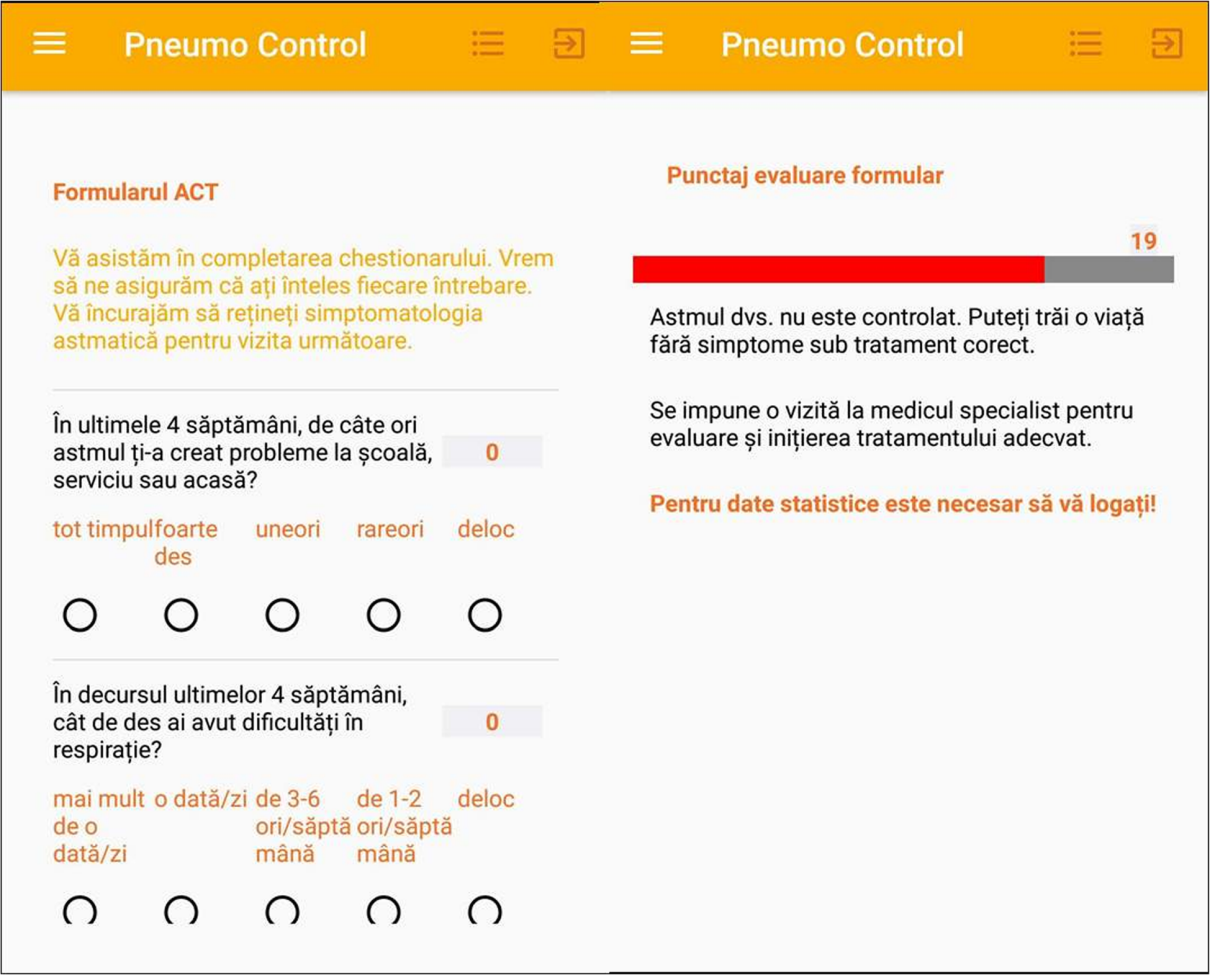
ACT questionnaire and assessment score. Formularul ACT – ACT questionnaire. Va asistam in completarea chestionarului. Vrem sa ne asiguram ca ati inteles fiecare intrebare. Va incurajam sa retineti simptomatologia astmatica pentru vizita urmatoare - We assist you in completing the questionnaire. We want to make sure you understand each question. We encourage you to remember the asthma symptomatology for your next visit. In ultimele 4 saptamani, de cate ori astmul ti-a creat probleme la scoala, serviciu sau acasa? – In the past 4 weeks, how much of the time did your asthma keep you from getting as much done at work, school or at home?. In decursul ultimelor 4 saptamni, cat de des ai avut dificultati in respiratie – During the past 4 weeks, how often have you had shortness of breath?

**Fig. 3:**
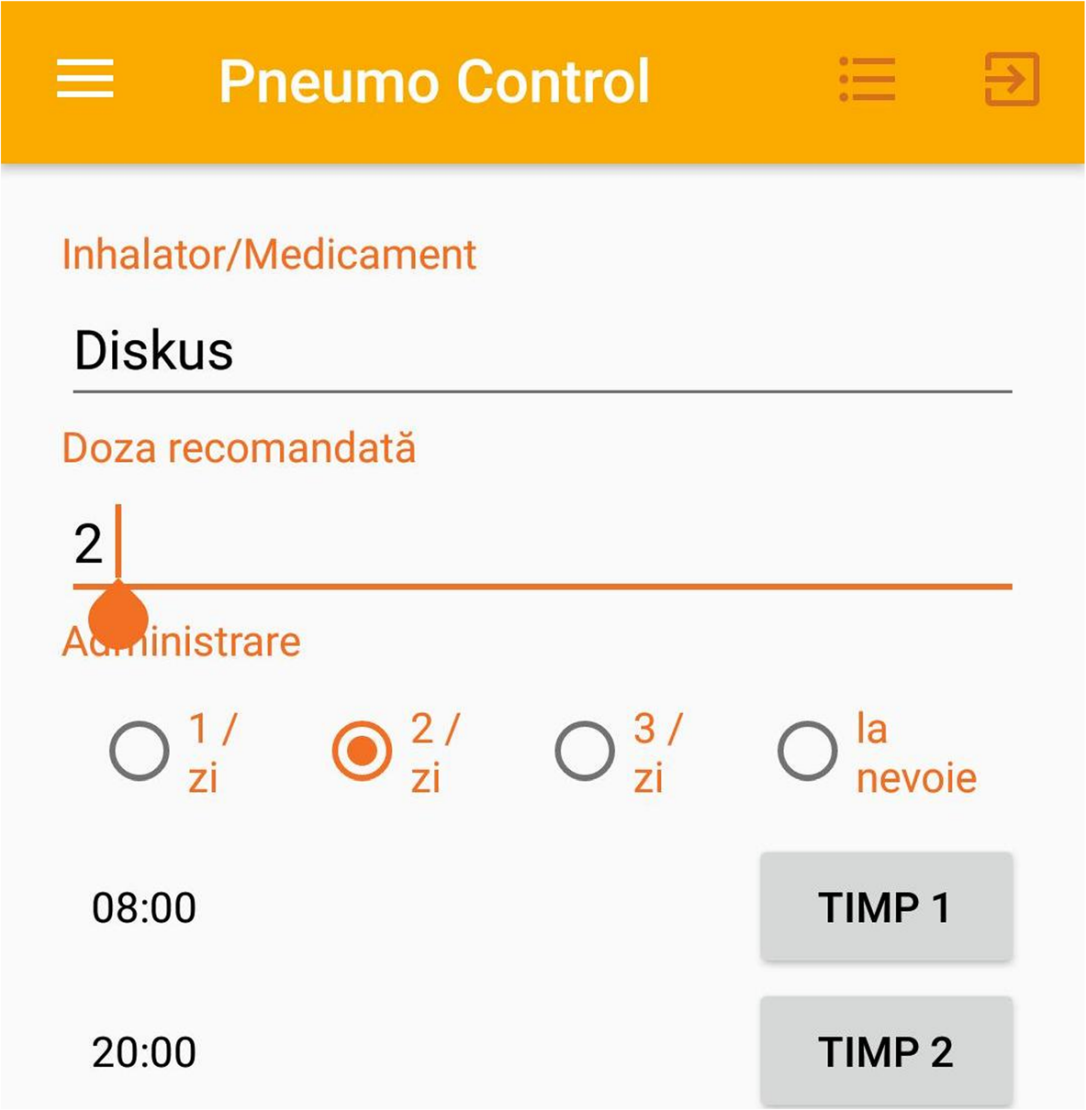
Self-management plan and prescribed medication. Inahaltor/Medicament – inhaler/medication. Doza recomandata – recommended dose. Administrare – administration

**Fig. 4:**
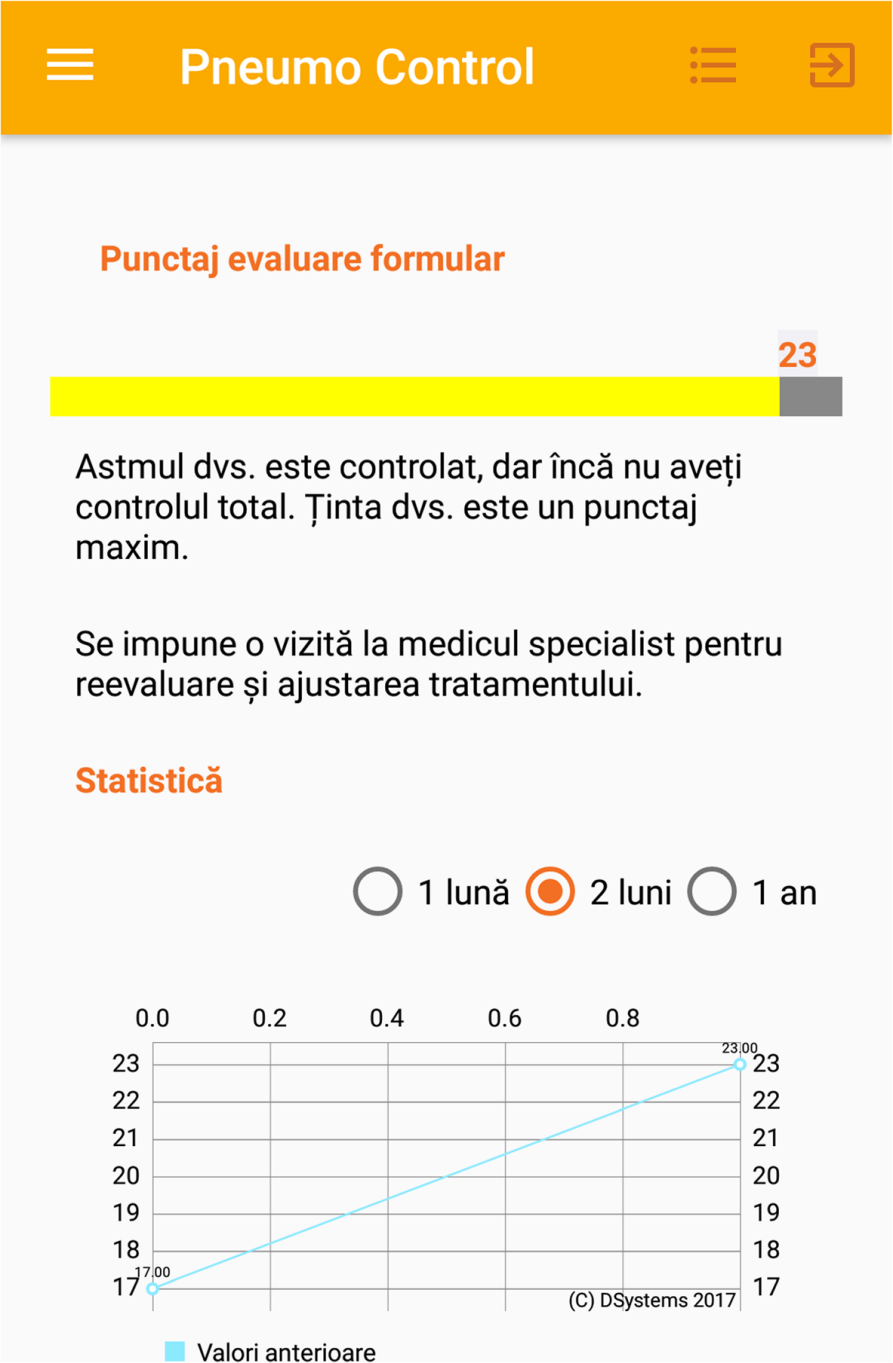
Evolution of ACT score. Puncatj evaluare formular – evaluation score form. Astmul dvs. Este controlat, dar încă nu aveți controlul total. Ținta dvs. Este un punctaj maxim – Your asthma is controlled but not totally. Your objective is a better score. Se impune o vizita la medical specialist pentru reevaluare si ajustarea tratamentului – you need to see a specialist doctor for reevaluation and medical treatment adjustment

This device (Figs. 1, 2, 3, 4) has been previously successfully teste in another study that analyzed the inhaler technique errors in Romanian patients with asthma [22]. The application contains different sections that helped the patients to improve adherence to treatment and self-management such as: pulmonary rehabilitation exercises, videos with inhalation devices techniques and specific questionnaires such as ACT.

### Statistical analysis

Data were analyzed using the SPSS v.17 software (SPSS Inc., Chicago, IL, USA). The graphical representations were generated using GraphPad Prism v8.0.2. A *p*-*value* of 0.05 was considered as the threshold for statistical significance, and a confidence level of 0.95 was considered for estimating intervals.

Measured data were described as mean (standard deviation) for numerical continuous variables with Gaussian distribution, median (percentile 25% - percentile 75%) for numerical variables without Gaussian distribution, or absolute frequency (percentage) for categorical variables. Continuous variable distributions were tested for normality using the Shapiro–Wilk’s test and for equality of variances by using Levene’s test.

The significance of the differences between the group of patients using the mobile app and the asthma control group was assessed using the Student’s *t*-test (means, Gaussian populations), Mann–Whitney *U* test (medians, non-Gaussian populations) or Kruskal-Wallis test (medians, non-Gaussian populations), and Pearson chi-square or Fisher’s exact test (proportions) were used. The significance of differences between the distribution of the with-in groups was assessed by applying the related-samples Friedman’s two-way analysis of variances by ranks, which is the non-parametric alternative to the one-way repeated measures ANOVA test. The related groups contained the same patients in each group, and each group is a repeated measurement of the same dependent variable. At the same time, we performed pairwise comparisons with a Bonferroni correction for multiple comparisons. The statistical significance was accepted at the adjusted *p-value* <  0.01 level.

## Results

The group of asthma patients who received only treatment, without using the smartphone app, included 54 patients (30 males and 24 females), aged between 18 and 72 years, mean age 38.59 (±17.64) years, 95% CI (33.78; 43.41) (Table 1). The median ACT score, at baseline, was 18.00 (17.00–19.00). Most of these patients (85.18%) used a reliever inhaler. In addition, more than a half of the patients (53.66%) presented severe exacerbations which required hospitalization, while 46.34% of the patients presented mild to moderate exacerbations, without hospitalization.

**Table 1:** Characteristics of patients at baseline

Parameters	Asthma patients (*N* = 54)	Asthma patients using mobile app (*N* = 39)	*p*-value^d^
Age (years)^a^	38.59 (±17.64)	36.87 (±15.44)	0.626
Gender (M)^b^	30 (55.6%)	21 (53.8%)	0.870
ACT score^c^	18.00 (17.00–19.00)	19.00 (18.00–20.00)	0.495
Reliever inhaler used ^b^	46 (85.18%)	39 (100%)	0.019
Exacerbations^b^	41 (75.9%)	39 (100%)	0.001
Required hospitalization	22 (53.66%)	23 (58.97%)	0.632
Did not require hospitalization	19 (46.34%)	16 (41.03%)	0.632

*Abbreviations: ACT* Asthma Control Test

^a^ Values are presented as mean (± st.dev.)

^b^ Values are presented as absolute frequency (percentage)

^c^ Values are presented as median (percentile 25% - percentile 75%)

^d^*p*-value was computed using independent samples t-Test for continuous variables with Gaussian distribution, Mann-Whitney U test for continuous variables with non-Gaussian distribution, or Pearson Chi-Square (or Fisher’s exact) test for nominal variables

The group of asthma patients who used the mobile app, in addition to their treatment, included 21 males and 18 females, aged between 18 and 66 years, mean age 36.87 (±15.44) years, 95% CI (31.87; 41.88) (Table 1). The median ACT score was 19.00 (18.00–20.00), being not significantly different than the ACT score of the control group (Mann-Whitney U test, *p* = 0.495). At baseline, all the patients used a reliever inhaler, significantly more than in the control group (Fisher’s exact test, *p* = 0.019). More than a half of the patients (58.97%) presented severe exacerbations requiring hospitalization, while 41.03% of the patients presented exacerbations which did not require hospitalization.

At baseline, all the patients of the group using the app presented exacerbations, while 85.18% of the patients of the control group not using the app presented exacerbations (chi-square test, X^2^ (1) = 10.915, *p* = 0.001). We observed that there were not significant differences concerning the number of exacerbations both requiring and not requiring hospitalization between the group of patients using the mobile app and the control group, 58.96% vs 53.66% (chi-square test, X^2^ (1) = 0.230, *p* = 0.632), for severe exacerbation, and 41.03% vs 46.34% (chi-square test, X^2^ (1) = 0.230, p = 0.632), for mild to moderate exacerbation.

We compared the ACT score, the number of reliever inhaler used, and number of exacerbations with both requiring and not requiring hospitalization for the group using the mobile app and the control group of asthma patients at the four evaluation moments. At the first evaluation (T_1_), after 3 months, the ACT score of patients using the mobile app was significantly higher than the score of asthma patients not using the app (Mann-Whitney U test, *p* <  0.001). Similarly, at the other evaluations (T_2_, T_3_ and T_4_), we observed that the ACT score was significantly higher in case of asthma patients using the app than the control group (Mann-Whitney U test, *p* <  0.001) (Table 2).

**Table 2:** Comparison of asthma patients vs. asthma patients using the mobile app

Parameters	Evaluation time	Asthma patients (*N* = 54)	Asthma patients using mobile app (*N* = 39)	*p*-value^c^
ACT score^a^	T _1_	19.00 (19.00–20.00)	21.00 (20.00–22.00)	< 0.001
	T_2_	20.00 (20.00–22.00)	23.00 (23.00–24.00)	< 0.001
	T_3_	21.00 (20.00–22.00)	23.00 (23.00–24.00)	< 0.001
	T_4_	21.00 (20.00–22.00)	23.00 (23.00–24.00)	< 0.001
Reliever inhaler used^b^	T _1_	40 (74.07%)	9 (23.08%)	< 0.001
	T_2_	21 (38.88%)	4 (10.26%)	0.002
	T_3_	14 (25.92)	1 (2.56%)	0.003
	T_4_	17 (31.48%)	4 (10.26%)	0.016
Exacerbations^b^	T _1_	25 (46.30%)	4 (10.30%)	< 0.001
	T_2_	8 (14.8%)	5 (12.8%)	0.784
	T_3_	9 (16.70%)	1 (2.60%)	0.041
	T_4_	13 (24.10%)	4 (10.30%)	0.089
Exacerbation with hospitalization^b^	T _1_	0 (0%)	0 (0%)	NA
	T_2_	1 (12.5%)	0 (0%)	0.615
	T_3_	1 (11.12%)	0 (0%)	0.900
	T_4_	5 (38.46%)	0 (0%)	0.261
Exacerbations without hospitalizations^b^	T _1_	25 (100%)	4 (100%)	NA
	T_2_	7 (87.5%%)	5 (100%)	0.615
	T_3_	8 (88.89%)	1 (100%)	0.900
	T_4_	8 (61.54%)	4 (100%)	0.261

*Abbreviations: T*_*1*_ evaluation at 3 months, *T*_*2*_ evaluation at 6 months, *T*_*3*_ evaluation at 9 months, *T*_*4*_ evaluation at 12 months. *ACT* Asthma Control Test

^a^ Values are presented as median (percentile 25% - percentile 75%)

^b^ Values are presented as absolute frequency (percentage)

^c^*p*-value was computed using the Mann-Whitney U test for continuous variables with non-Gaussian distribution or Pearson Chi-Square (or Fisher’s exact) test for nominal variables

We also observed significant differences between the ACT score of the patients group using the mobile app and the group using only the treatment stratified by gender. The ACT-T_1_ scores for men patients who did not use the mobile app vs. who used the mobile app were 19.00 (19.00–20.00) vs 21.00 (20.00–22.00), *p* < 0.001, and in case of women patients, the scores were 20.00 (19.00–20.00) vs. 22.00 (21.00–23.00), *p* < 0.001. The ACT-T_2_ for men patients who did not use the mobile app vs. who used the mobile app were 22.00 (20.00–22.00) vs 23.00 (23.00–24.00), *p* < 0.001, and in case of women patients, the scores were 20.00 (20.00–22.00) vs. 23.50 (23.00–24.00), *p* < 0.001. The ACT-T_3_ scores of men patients who did not use the mobile app vs. who used the mobile app, 22.00 (20.00–22.00) vs 23.00 (23.00–24.00), *p* < 0.001, and in case of women patients, the scores were 21.00 (20.00–22.00) vs. 23.50 (23.00–24.00), *p* < 0.001. Also, we observed significant differences between ACT-T_4_ scores of men patients who did not use the mobile app vs. who used the mobile app, 21.00 (20.00–22.00) vs 24.00 (23.00–24.00), *p* < 0.001, as well as for women patients, 21.00 (19.50–22.00) vs. 23.00 (23.00–24.00), *p* < 0.001.

When comparing the number of reliever inhaler used between the two groups, we observed significant differences at the first evaluation (T_1_; Pearson Chi-Square X^2^ (1) = 23.626, *p* < 0.001), at the second evaluation (T_2_; Pearson Chi-Square X^2^ (1) = 9.445, *p* = 0.002), at the third evaluation (T_3_; Pearson Chi-Square X^2^ (1) = 9.136, *p* = 0.003), as well as the fourth evaluation (T_4_; Pearson Chi-Square X^2^ (1) = 5.836, *p* = 0.016).

Considering the number of exacerbations, at the first evaluation T_1_, we observed that significantly less patients using the app presented exacerbations, 10.30% vs. 46.30% (Pearson Chi-square test, X^2^ (1) = 13.707, *p* < 0.001). We did not observe exacerbations requiring hospitalization in any of the two groups, while the number of exacerbations which did not require hospitalization was significantly higher in case of the first group, when comparing to the second group (Table 2).

At the second evaluation, we observed no significant differences between the number of exacerbations between the two groups, 12.8% vs. 14.8% (Pearson Chi-square test, X^2^ (1) = 0.075, *p* = 0.784). In addition, we observed one exacerbation requiring hospitalization in the asthma patients’ group and still zero in the group using the application. When considering the third evaluation, we observed that in the group using the app were significantly less patients with exacerbations, 2.6% vs. 16.7% (Fisher’s exact test, *p* = 0.041). Also, in the group of patients using the app, none of the patients presented exacerbations requiring hospitalization, while in the group not using the app, 11.12% of patients presented exacerbations requiring hospitalization. At the fourth evaluation, there were not significant differences between the groups of patients using the application vs. patients not using the app, 10.3% vs. 24.1% (Pearson Chi-square test, X^2^ (1) = 2.894, *p* = 0.089). Also, in the group of patients using the app, none of the patients presented exacerbations requiring hospitalization, while in the group not using the app, 38.46% of patients presented exacerbations requiring hospitalization.

**Fig. 5:**
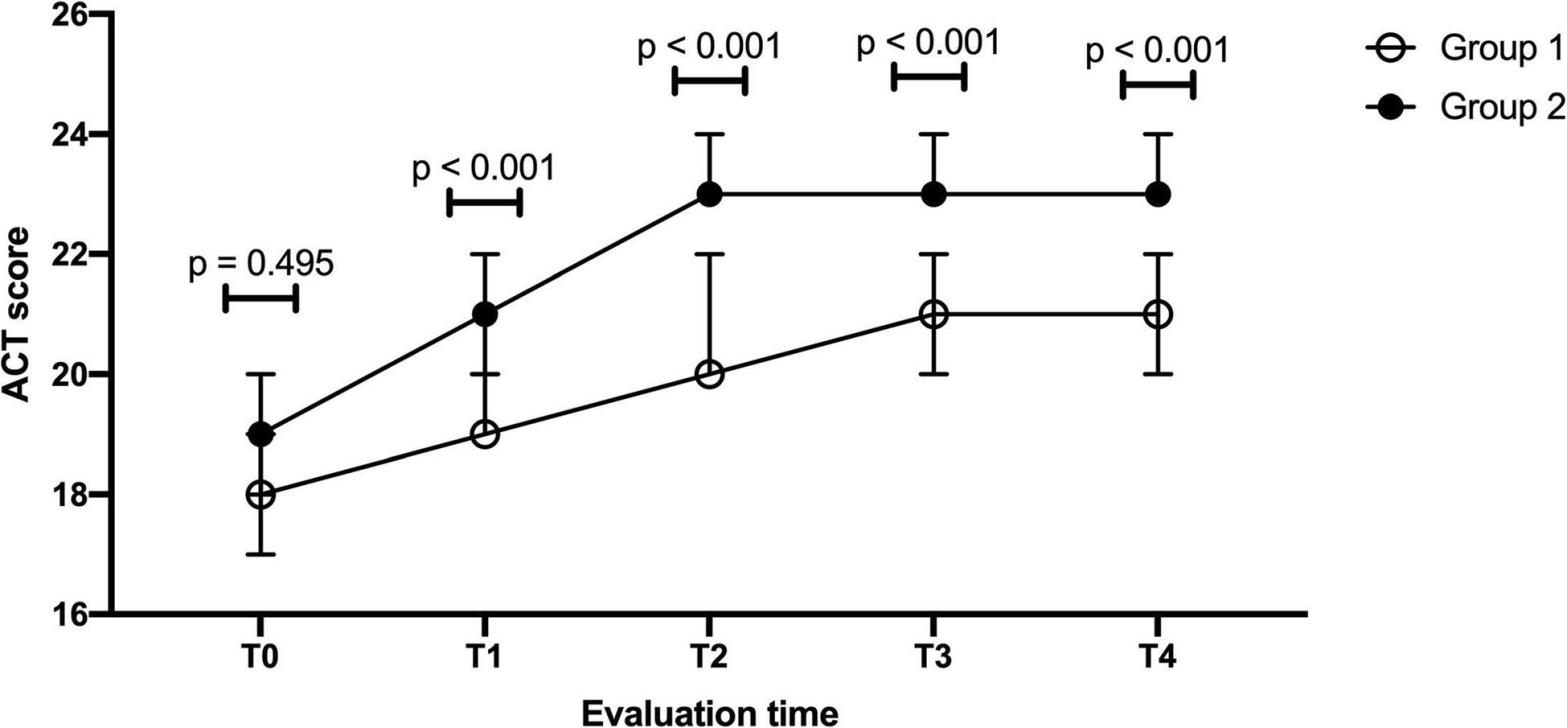
The ACT score of the asthma patients (Group 1) and the asthma patients using the mobile application (Group 2). (T_0_ = baseline evaluation, T_1_ = evaluation at 3 months; T_2_ = evaluation at 6 months; T_3_ = evaluation at 9 months; T_4_ = evaluation at 12 months. ACT = Asthma Control Test)

Figure 5 illustrates the ACT score at different evaluation moments for both asthma patients’ group and the group of asthma patients using the mobile app.

When comparing the evolution of the ACT score with-in each group, we observed significant differences between the five measurements (related-samples Friedman’s test, X^2^ (4) = 67.643, *p* < 0.001), in case of asthma patients which did not use the app. But after performing pairwise comparisons with a Bonferroni correction, we observed that ACT score was significantly lower at baseline than the other evaluation moments (ACT-baseline vs. ACT-T_1_, *p* = 0.035; ACT-baseline vs. ACT-T_2_, *p* < 0.001; ACT-baseline vs. ACT-T_3_, *p* < 0.001; ACT-baseline vs. ACT-T_4_, *p* < 0.001). At the same time, we observed that the score at the second, third and fourth evaluations were significantly higher than the score at the first evaluation (ACT-T_1_ vs. ACT-T_2_, *p* = 0.038; ACT-T_1_ vs. ACT-T_3_, *p* = 0.004; ACT-T_1_ vs. ACT-T_4_, *p* = 0.009). On the contrary, when comparing the ACT score at second evaluation with the score at the third and fourth evaluation as well as the score at the third evaluation with the fourth evaluation, we did not observe significant differences. The considered *p*-value after being adjusted by the Bonferroni correction was 0.01.

In case of asthma patients using the app, we observed that the ACT score was significantly different between the five measurements (related-samples Friedman’s test, X^2^ (4) = 112.787, *p* < 0.001). After performing pairwise comparisons with a Bonferroni correction, we observed that ACT score was significantly lower at baseline than the other evaluation moments (ACT-baseline vs. ACT-T_1_, *p* = 0.003; ACT-baseline vs. ACT-T_2_, *p* < 0.001; ACT-baseline vs. ACT-T_3_, *p* < 0.001; ACT-baseline vs. ACT-T_4_, *p* < 0.001). At the same time, we observed that the score at the second, third and fourth evaluations were significantly higher than the score at the first evaluation (ACT-T_1_ vs. ACT-T_2_, *p* = 0.001; ACT-T_1_ vs. ACT-T_3_, *p* < 0.001; ACT-T_1_ vs. ACT-T_4_, *p* < 0.001). On the contrary, when comparing the ACT score at second evaluation with the score at the third and fourth evaluation as well as the score at the third evaluation with the fourth evaluation, we did not observe significant differences.

## Discussion

In this study we analysed if a mobile app specially designed for patients with asthma in Romanian population can improve self-management and disease control.

Although in the last years many studies have been published regarding the feasibility and importance of providing self-management and control in patients with asthma, there are a large number of studies that found no significant improvement regarding symptoms and asthma control after using m-health app [23–26].

The present study showed that self-management has been improved and exacerbation rate has been significantly reduced after 3 months of using the specific mobile phone app compared to the group that did not used the app. Our findings could be explained by the fact that those who used the app had videos to remind them the correct inhalation technique; pulmonary rehabilitation exercises and action plans.

A recent systematic review that analysed patient self-management of asthma using mobile app found that from the researched papers, 90% were indicative of significant impact on most outcomes evaluated [27], and just one paper, although improvements were seen, mobile phone app were not superior to paper recording [16].

Another study that analyzed the impact of a mobile app on health outcomes in patients with asthma found that the patients that used the app achieved a well-controlled asthma score (49%) (ACT> 19) compared to the control group (27%), values that were statistically significant. Furthermore, patients using the app improved their ACT score by 6 points, whereas the control group had an improvement by only 2 points (median) [14].

Compared to these results, ACT improvements were lower in our study. However, both groups improved their scores over the one-year study period. Participants using the app showed the highest ACT scores. Moreover, we observed that the mean score of the group that used the mobile phone app significantly improved when comparing with the initial evaluation.

We also observed that after the third evaluation, although patients did not reach the maximum ACT score, no more significant improvements were registered in both groups.

Cook et al. observed that a minimally proactive use of a mobile app had a significantly improvement in asthma control in patients that previously had uncontrolled asthma. They reached this conclusion in only 4 months. Moreover, the authors emphasized that the use of the app was associated with a statistical and clinical improvement in ACT scores [12]. Results demonstrated an increase of 3 points in the mean ACT score after only 5 weeks of app use. The patients with uncontrolled asthma (an ACT score under 20 points) benefited the most, with an mean increase of 5.7 points [12].

Although it have been suggested that self-management effectiveness is reduced when assessed over long-term follow-up [28], this study showed that patients had a good compliance and asthma control has been improved in a period over 1 year. A possible explanation for this situation could be the fact that the patients knew they were in a study and that they had to come for re-evaluation every 3 months, while in the study of Kauppinen et al., the patients were assessed for a much longer period (> 10 years).

A study that evaluated web-based asthma self-management found that emergency rooms or acute visits to the physician for asthma symptoms did not significantly change in the studied group [29]. Compared with this study we found that the use of action plans from the app and the step up self-medication recommended by the device, had a significant impact on the physician and emergency room visits.

Two studies analysed the impact of mobile phone based self-management on unscheduled visits to the emergency department and hospital admissions due to asthma-related complications [15, 16]. While one study showed that the patients that used the mobile phone app had a reduced attendance to the emergency department compared to the control group another study found no significant outcomes regarding the emergency department visits [16].

Regarding the hospital admissions, the results of both authors were statistically non-significant. In the present study, we observed a significant reduction in hospital admission for the group using the app, more exactly, starting from the second evaluation, there were no exacerbations requiring hospitalization. When considering the exacerbations that did not require hospitalization, we observed significantly lower numbers in the group using the app for the second, third and fourth evaluations.

A limitation of this study could be the small sample size. Another limitation is that compared to other studies that included younger or older patients, we assessed patients over 18 years, with a mean age of 38.59 (±17.64) years, at this age it can be considered that the patients are more likely to use self-management app especially, due to the awareness of the disease. Because we did not use a randomized design there are a number of factors that could have influenced to the decision to use the app. These factors could have affected the patients’ self-management and thus the asthma control and related outcomes. This app was designed especially for the Romanian population and is restricted to this area thus the app cannot be used in other countries. Also, it has to be noted that due to the selection method and lack of randomization, a selection bias is possible and the fact that the patients that used the app could have been more motivated than the control group.

## Conclusion

Our study indicates that smartphone apps are an effective way to improve asthma control and self-management when used continually in our population. We found significant positive effects in disease control and exacerbation frequency. Pneumocontrol is a potential app that can be useful in order to improve asthma outcomes in our population.

## Data Availability

The authors confirm that all data underlying the findings are fully available without restriction. Since the database with the analyzed data contains personal patients’ information, the data will be available for all interested researchers after submitting a request to the ethics committee of the hospital.

## Abbreviations

GINA: Global Initiative for Asthma
FEV_1_: Forced Expiratory Volume
FVC: Forced Vital Capacity
ACT: Asthma Control Test
COPD: Chronic Obstructive Pulmonary Disease
T_0_: First evaluation at baseline
T_1_: Evaluation at 3 months
T_2_: Evaluation at 6 months
T_3_: Evaluation at 9 months
T_4_: Evaluation at 12 months
